# Landauer-Based Economic Temperature in Blockspace Markets: Evidence from Bitcoin and Ethereum

**DOI:** 10.3390/e28050508

**Published:** 2026-05-01

**Authors:** Michael Zouari, Ilan Alon, Zeev Shtudiner

**Affiliations:** 1 Department of Economics and Business Administration, Ariel University, Ariel 40700, Israel; ilan.alon@uni-corvinus.hu (I.A.); zeevs@ariel.ac.il (Z.S.); 2 Department of Strategic Management, Institute of Strategy and Management, Corvinus University, Fővám tér 8, 1093 Budapest, Hungary

**Keywords:** Landauer principle, economic temperature, blockspace markets, Bitcoin, Ethereum, econophysics, Carnot efficiency, structural breaks

## Abstract

The Landauer principle motivates the definition of economic temperature as the monetary price of processing a bit irreversibly. No empirical test of this definition exists in transparent fee markets. This paper fills that gap using daily Bitcoin and Ethereum data, constructing canonical thermodynamic state variables and evaluating five diagnostic layers: state variable behavior, Maxwell-type integrability, Carnot-style efficiency bounds, nonlinear regime separation, and structural break sensitivity to protocol events. Bitcoin’s log-temperature behaves as a persistent mean-reverting process with an AR(1) coefficient of 0.97 and a half-life of 21 days; Ethereum is highly persistent, with weaker formal evidence of stationarity than Bitcoin. Maxwell integrability is frequency-dependent: Bitcoin passes all four relations at monthly frequency, whereas Ethereum passes two of four. Carnot-style evidence is the strongest: realized fee extraction efficiency stays well below the implied bound, with daily compliance exceeding 97% on both chains. Structural breaks around Bitcoin ordinals, EIP-1559, the merge, and Shanghai confirm that protocol changes reorganize the temperature relation. The thermodynamic framework provides structure that standard fee market analysis does not, including a first principles efficiency bound and a state space coherence test. The findings provide partial, frequency-dependent, and chain-specific empirical support for a Landauer-based thermodynamic description of blockspace markets.

## 1. Introduction

Thermodynamic language becomes scientifically useful in economics only when it does more than decorate intuition. Modern thermodynamics must explicitly incorporate information, because erasure, copying, and feedback carry measurable entropic and energetic costs [1]. When interacting systems exchange information alongside energy, the entropy balance of a subsystem acquires an information flow term that standard energy accounting alone cannot capture [2]. This logic extends to nonequilibrium settings, where work, heat, and entropy production can be formulated at the level of fluctuating trajectories [3] and where retaining nonpredictive information is itself thermodynamically costly because dissipation rises whenever a system stores information that does not improve prediction [4]. These results confirm that information is not external to thermodynamics but integral to it.

This informational foundation carries direct consequences for econophysics. Yakovenko & Rosser [5] define the field as the application of statistical physics methods to large economic systems, emphasizing quantitative regularities in money, wealth, and income distributions instead of representative-agent abstraction. Bouchaud & Mézard [6] demonstrate the approach in practice: stochastic exchange and speculative dynamics generate broad wealth distributions and even concentration regimes. Bouchaud [7] sharpens the methodological point by arguing that when data conflict with a model, the model should be amended or discarded, and that economics should be disciplined by empirical evidence capable of handling “wild” markets rather than shielded by elegant axioms. Together, these contributions set the standard for the present study: thermodynamic concepts such as temperature, entropy, and dissipation matter scientifically in economic systems only if they yield observable structure and potentially falsifiable regularities.

Within the econophysics tradition described above, Bormashenko & Shendrik [8] introduce a sharper definition. They distinguish between marginal economic temperature, defined as the minimal price of processing one bit of information irreversibly, and actual economic temperature, which captures what real economic agents pay per bit in practice. Their framework points to settings where information processing carries explicit, measurable costs, including high-frequency trading, electronic markets, and ledger-based financial systems, as the most promising empirical domain.

This redefinition is grounded in the Landauer tradition. The Landauer principle sets a lower theoretical limit on the energy cost of computation: any irreversible informational change dissipates at least kBTln2 per bit [9]. Temperature thereby acquires a fundamental informational meaning as the sole physical quantity governing the cost of isothermal bit erasure [10], and the bound holds universally, remaining unchanged even in many-valued logical systems [11]. Logical irreversibility and thermodynamic reversibility are not identical: logically irreversible erasure can still be implemented in a thermodynamically reversible way in the quasi-static limit [12].

Blockspace markets are an unusually strong empirical laboratory because they allocate a scarce information-processing resource in public view. Easley et al. [13] show that Bitcoin transaction fees emerged as the blockchain evolved from a mining-based structure toward a market-based ecology, connecting fees directly to waiting times, user participation, and exogenous structural constraints embedded in the protocol. Huberman et al. [14] formalize this congestion channel: users pay fees to gain priority and avoid delays, no delays imply no fees, and the congestion–fee relation becomes highly nonlinear as blocks approach capacity. Tsang & Yang [15] provide direct empirical support by showing that both transaction fees and transaction volume rise when the network is congested, while also stressing that fluctuating fees impose an additional uncertainty cost on users.

These results matter because they show that blockspace markets do not merely host transactions; they visibly price ledger writing, queue priority, and throughput scarcity. On the pricing side, Biais et al. [16] argue that the fundamental value of Bitcoin depends on a stream of net transactional benefits, while the relevant costs include the fees paid to have transactions mined along with limited convertibility and exchange frictions. On the settlement side, Saleh [17] shows that a blockchain’s usefulness depends on validators agreeing on the ledger, because settlement requires all copies to record the transaction consistently, and that in proof-of-stake systems the incentive structure differs precisely because validators are also stakeholders.

For that reason, Bitcoin and Ethereum are natural first test beds for a Landauer-based empirical design. They preserve the core economic problem of distributed settlement while making fees, congestion, throughput, and protocol design unusually observable. Blockspace markets are not metaphorically informational; they are markets in which information-processing costs are directly measurable.

Recent research has moved thermodynamic language closer to observable markets. Li et al. [18] treat the limit order book as a thermodynamic system using high-frequency Bitcoin spot data, deriving market temperature and market entropy from kinetic and potential energies. They show that market temperature behaves as a robust liquidity indicator, while market entropy captures disorder relevant to instantaneous price volatility. Zhong et al. [19] take a different approach, defining transaction entropy as a measure of uncertainty in making successful transactions. They report that it is lowest at equilibrium, decreases in shortage markets, increases in surplus markets, and is reduced by centralized price filtering.

Li et al. [18] analyze thermodynamic measures in a centralized order book and Zhong et al. [19] develop an entropy metric for transactional uncertainty; by contrast, Bormashenko & Shendrik [8] define economic temperature in Landauer terms as a cost of irreversibly processed information. That distinction motivates the present study.

The theoretical economics of blockchains is already substantial, but organized around different questions. Biais et al. [20] model proof-of-work blockchains as coordination games in which forks arise through strategic complementarities, information delays, and software upgrades. Cong & He [21] show that blockchain expands the contracting space through smart contracts while the information distribution required for consensus may foster collusion. At the market level, Makarov & Schoar [22] document large and recurrent arbitrage opportunities across exchanges, with cross-country deviations far exceeding within-country deviations, and Cong et al. [23] show that tokens derive value from enabling platform transactions through network effects.

The market microstructure literature, meanwhile, already treats modern trading as an information-processing environment. Algorithmic decision-making, low latency, and high message rates define high-frequency trading [24], and increased algorithmic activity has been shown to improve liquidity and quote informativeness [25]. At the venue level, Menkveld [26] characterizes a major high-frequency trader as a modern multi-venue market maker, while Brogaard et al. [27] show that high-frequency traders contribute to price discovery by acting with permanent price movements and countering transitory pricing errors. Yet neither this literature nor the blockchain economics reviewed above test whether a fee-based temperature mapping yields coherent empirical regularities in blockspace markets.

Accordingly, the central research question of this paper is straightforward: do blockspace markets exhibit coherent empirical regularities when economic temperature is operationalized as fee-based irreversible information cost?

The five predictions form a diagnostic hierarchy: H1 tests whether temperature is a meaningful state variable, H2 and H3 test whether it generates coherent state space structure and efficiency constraints, and H4 and H5 test whether it responds to economic regimes and architectural changes.

The empirical design yields five predictions:

**H1.**  *Temperature should behave more like a bounded state variable in Bitcoin than in Ethereum, because Bitcoin presents the cleaner fee structure [13,14].*

**H2.**  *Maxwell-type consistency should strengthen when daily observations are aggregated to weekly and monthly frequency, because microstructure noise should mask rather than create state space regularity [1,8].*

**H3.**  *Realized fee extraction efficiency should remain below Carnot-style bounds on both chains [8].*

**H4.**  *Temperature distributions should separate across market regimes and congestion tiers, because network pressure alters the economic cost of prioritized settlement [13,15].*

**H5.**  *Major protocol events should induce structural breaks in the batching–temperature relation, because protocol change alters the rules governing settlement and information processing [28,29].*

The contribution is threefold. First, the paper provides an empirical operationalization of a recent Landauer-based theory rather than a conceptual analogy. Second, it uses a multi-diagnostic design in which the theory is evaluated through several mutually informative tests. Third, it seeks evidence on boundary conditions, identifying where the thermodynamic interpretation is stronger, where it is weaker, and why that asymmetry matters.

## 2. Materials and Methods

### 2.1. Research Design and Case Selection

This study adopts a comparative two-chain design, but not as a symmetric comparison. Bitcoin is the primary empirical case because its fee market visibly prices congestion under protocol-imposed structural constraints [13,14,15], making it the cleaner canonical setting for a Landauer-based temperature test. Ethereum is treated as a secondary empirical case. It preserves explicit blockspace pricing, but under a different architectural logic; Biais et al. [16] emphasize that cryptocurrencies derive value from transactional benefits including fees, while Saleh [17] shows that in proof-of-stake systems the incentive structure differs because validators are also stakeholders. Analytically, Ethereum is valuable precisely because it tests how far the temperature mapping travels beyond Bitcoin’s cleaner fee queue.

### 2.2. Data, Sampling, and Canonical Specification

This paper uses one canonical specification throughout. The Bitcoin daily clean sample contains 3371 observations (1 January 2017 to 28 March 2026), while the Ethereum daily clean sample contains 3887 observations (8 August 2015 to 29 March 2026). Weekly and monthly panels are constructed from the same canonical state variables by direct mean aggregation and not by redefining the variables or introducing alternative proxies at lower frequencies. All code, data, and outputs are archived in a frozen replication package. Bitcoin on-chain data were obtained from Blockchain.com (Blockchain Luxembourg S.A., Luxembourg City, Luxembourg), Google BigQuery (Google LLC, Mountain View, CA, USA), and Mempool.space (open-source project, https://mempool.space, accessed on 20 March 2026); Ethereum on-chain data were obtained from Google BigQuery (Google LLC, Mountain View, CA, USA).

A single fixed mapping is used throughout to prevent specification drift across diagnostic layers. The weekly panel is therefore generated by direct mean aggregation of the canonical state variables on a Monday-anchored week, and the monthly panel by direct mean aggregation on calendar months. The regime partitions in Section 2.7 and Section 2.8 use auxiliary identification variables (mempool size, transaction count, fee per transaction), not the canonical log-transformed state variables. For Bitcoin, the partitioning variable (mempool size) is not an ingredient of T*. For Ethereum, fee per transaction is a component of T*, introducing some mechanical dependence; this is discussed in Appendix B.

### 2.3. Deriving the Thermodynamic State Space from Landauer Cost

The thermodynamic structure is derived as a restricted empirical representation of fee-based irreversible information processing in public settlement systems, not imposed as a metaphorical overlay. The starting point is the Landauer-based definition of actual economic temperature:

$${E_{t}}^{\mathrm{act}}={T_{t}}^{*}\cdot ln2,$$

where ${E_{t}}^{\mathrm{act}}$ is the realized monetary cost of irreversibly processing one bit and ${T_{t}}^{*}$ is the corresponding actual (as distinct from marginal) economic temperature in the sense of Bormashenko & Shendrik [8]. This distinction is central here because blockchain markets operate far above the physical Landauer limit; the empirical object is the realized market cost of irreversible ledger inclusion, not the physical minimum.

In a public blockchain, the irreversible economic operation is confirmed ledger inclusion: a transaction is selected, ordered, validated, and written into the replicated state, after which reversal requires a compensating transaction or a chain reorganization. Transaction fees therefore provide an observable monetary cost of this irreversible information processing. The empirical temperature variable is built from the user-paid confirmation cost and taken in log form:

$$T_{t}=ln\left({T_{t}}^{*}\right),$$

because the level cost is positive, highly skewed, and multiplicative in the fee and asset-price components.

The thermodynamic state space has four coarse-grained coordinates:

(T,S,P,V).

Here, T is the fee-based economic temperature, S is a state complexity coordinate, P is a congestion/demand-pressure coordinate, and V is executed settlement volume. These are empirical coordinates of a fee-based information-processing system, not literal physical temperature, entropy, pressure, and volume. The testable claim is narrower: if the four coordinates approximate a coherent thermodynamic state, their cross-partial relations should be approximately integrable. Table 1 summarizes the notation used throughout.

The primitive economic potential is a latent scalar function of the extensive state variables S and V:

U=U(S,V).

U is the scalar resource potential required to maintain a ledger state of complexity S while executing settlement volume *V* under a given protocol architecture. It is not market capitalization, aggregate utility, miner or validator revenue, or physical internal energy. Its empirical role is structural: the derivatives of U, not U itself, are observed through the mapped state variables.

Using the standard thermodynamic sign convention, its differential is

dU=T dS−P dV.

Therefore,

$$T={\left(\frac{\partial U}{\partial S}\right)}_{V},\;and\;P=-{\left(\frac{\partial U}{\partial V}\right)}_{S}.$$

T is the marginal fee-based cost of increasing state complexity at fixed volume; P is the marginal congestion force of increasing settlement volume at fixed complexity. Since U is latent, it is not estimated directly; this paper tests whether the observable variables behave as if such a smooth scalar potential exists.

The primitive potential is specified as U(S, V) rather than an open-system form U(S, V, N). This is a deliberate coarse-graining choice. Public blockchains are open transaction-processing systems, but the empirical object here is the daily realized settlement state after transactions have entered, competed for inclusion, and been confirmed, not the microscopic inflow of each user or order.

The transaction-flow dimension enters in two ways. First, executed settlement volume *V* captures the realized flow of blockspace or computational throughput. Second, the state complexity coordinate *S* absorbs part of the active user and transaction count dimension, because higher transaction activity and participation expand the economically relevant information maintained on the ledger. A full open-system model could introduce a chemical potential-like term,

dU=T dS−P dV+μ dN,

where N represents active users, addresses, or transaction arrivals. However, such a specification would require an identified conjugate variable μ, which is not observable at daily frequency. This paper therefore tests the minimal identifiable state representation. This restriction makes the framework more falsifiable: if the reduced (T, S, P, V) structure fails, the failure identifies where additional open-system channels are required.

The Legendre transforms of U generate the remaining potential functions:

H(S,P)=U+PV,  with dH=T dS+V dP,

F(T,V)=U−TS,  with dF=−S dT−P dV,

G(T,P)=U−TS+PV,  with dG=−S dT+V dP.

These potentials provide alternative descriptions of the same restricted state space. U is the primitive settlement potential; H is a congestion-adjusted settlement potential; F is a temperature-adjusted variant; and G is adjusted on both. If these potentials exist and are sufficiently smooth, equality of mixed partial derivatives implies four Maxwell-type restrictions:

$$MR1:{\left(\frac{\partial T}{\partial V}\right)}_{S}=-{\left(\frac{\partial P}{\partial S}\right)}_{V},$$

$$MR2:{\left(\frac{\partial T}{\partial P}\right)}_{S}={\left(\frac{\partial V}{\partial S}\right)}_{P},$$

$$MR3:{\left(\frac{\partial S}{\partial V}\right)}_{T}={\left(\frac{\partial P}{\partial T}\right)}_{V},$$

$$MR4:{\left(\frac{\partial S}{\partial P}\right)}_{T}=-{\left(\frac{\partial V}{\partial T}\right)}_{P}.$$

The Maxwell block is therefore an integrability diagnostic: it does not assume blockchain markets are literal thermodynamic systems but asks whether the mapped variables behave as if they are local derivatives of a common latent settlement potential. If those variables are merely correlated market observables, the cross-partial restrictions have no reason to hold. If they approximate a coherent thermodynamic state space, the restrictions should tighten under weekly and monthly aggregation, as high-frequency noise is averaged out. Rejections are also informative: they identify where the thermodynamic representation fails, is frequency-dependent, or is chain-specific.

The empirical mapping is intentionally chain-specific. Bitcoin and Ethereum do not expose the same economic object under different labels: they implement different settlement architectures. Bitcoin prices scarce byte-oriented blockspace through a public fee queue. Ethereum prices execution, computation, and state access through gas and, after EIP-1559, splits user-paid fees into burned base fees and validator tips. This paper therefore does not compare absolute temperature levels across chains: it compares diagnostic structure: persistence, integrability, Carnot compliance, nonlinear regimes, and protocol-break sensitivity.

For Bitcoin, the fee-based temperature proxy uses the fee rate quoted in satoshis per virtual byte. The level cost per bit is

$$E_{t}^{act,BTC}=\frac{{fee\_rate}_{t}\cdot P_{t}^{BTC}}{8\cdot 10^{8}},$$

where ${fee\_rate}_{t}$ is the daily median fee rate in satoshis per virtual byte, $P_{t}^{BTC}$ is the daily Bitcoin price in USD, 8 converts bytes to bits, and $10^{8}$ converts satoshis to BTC. The corresponding level and log-temperatures are

$$T_{t}^{*,BTC}=\frac{E_{t}^{act,BTC}}{ln2},\;and\;T_{t}^{BTC}=ln\left(T_{t}^{*,BTC}\right).$$

For Ethereum, the canonical temperature proxy is the frozen fee-per-transaction specification:

$$T_{t}^{ETH}=ln\left[\frac{{fee\_per\_tx}_{t}^{gwei}\cdot P_{t}^{ETH}}{8\cdot ln2}\right].$$

This proxy should be read as an effective user-paid settlement cost temperature, not as a literal byte-equivalent measure. The gwei-to-ETH conversion is a multiplicative constant in the level proxy and an additive constant in log-temperature; it drops out of Maxwell diagnostics and cancels in Carnot-style ratios. This paper therefore avoids cross-chain comparisons of absolute temperature levels. Gas-standardized Ethereum proxies serve as robustness specifications (see Table S4), since gas is Ethereum’s native computational pricing unit.

The remaining state variables follow the same structural logic. Bitcoin’s S proxy is the UTXO count, P is public mempool size, and V is average block size. Ethereum’s S proxy is transaction count, P is active addresses, and V is block count. These proxies are empirically tractable coordinates of state complexity, demand pressure, and realized settlement scale. Table 2 summarizes the canonical mapping.

### 2.4. Diagnostic Architecture

The empirical strategy is layered because no single test can establish coherent empirical regularities. Building on standard econometric tools for unit root testing [30], stationarity testing [31], and structural break estimation [32], this paper evaluates the fee-based temperature mapping through five complementary diagnostic layers: (1) state variable diagnostics, (2) Maxwell-type integrability tests, (3) Carnot-style efficiency constraints, (4) nonlinear regime and threshold analysis, and (5) structural break analysis around major protocol events.

### 2.5. State Variable Diagnostics

The state variable block combines four diagnostics. The ADF test [30] evaluates the null of a unit root, while the KPSS test [31] evaluates the null of stationarity. The two tests are used jointly because their nulls differ and therefore give a more disciplined reading than either test alone. In addition, an AR(1) persistence coefficient is estimated for the temperature series, and, when 0 < phi < 1, a half-life is computed as log(0.5)/log(phi). The target is not textbook stationarity but whether temperature shows visible mean reversion rather than uncontained drift.

### 2.6. Maxwell-Type Tests

The Maxwell block examines whether paired cross-partial restrictions emerge from the empirical state space. For each relation, the relevant partial derivatives are estimated using a binning-based within-bin slope procedure. The variable held “constant” is partitioned into quantile bins, and within each bin a local slope is estimated between the target and driver variables. These within-bin slopes are then averaged to obtain the partial derivative estimate. All frequencies use 10 quantile bins, matching the frozen specification.

The paired left-hand side and right-hand side estimates are then compared using a z-statistic built from the difference in estimated slopes and the associated cross-bin standard errors. A relation is treated as supported when the equality restriction is not rejected at the 5% level. The test asks whether approximate integrability emerges once high-frequency noise is reduced by temporal aggregation.

### 2.7. Carnot-Style Diagnostics

The Carnot block operates on the level temperature proxy T* (before the log transformation). For Bitcoin, high-congestion and low-congestion regimes are defined by fixed mempool size thresholds: days with mempool exceeding 100 MB are classified as hot, and days with mempool below 10 MB as cold. For Ethereum, high- and low-fee regimes are defined by a median split on the daily fee per transaction. From these regimes, an implied Carnot-style upper bound is computed as

$$\eta_{C}=1-\frac{T_{cold}}{T_{hot}}.$$

Realized efficiency is then measured chain by chain. For Bitcoin, actual efficiency is defined as transaction fees as a share of miners’ total revenue. For Ethereum, actual efficiency is defined as transaction fees as a share of total protocol-side revenue, that is, fees plus issuance, both expressed in USD. Daily compliance is the share of valid days on which realized efficiency does not exceed the implied bound. A monthly heat engine analog then correlates fee revenue with ΔT = T_month_ − T_cold_, asking whether larger temperature gradients are associated with greater extracted revenue.

### 2.8. Regime and Structural Break Procedures

Regime classification is handled in three steps. First, bull and bear states are defined by whether price is above or below its 200-day moving average. Second, congestion tiers are defined by fixed mempool size thresholds for Bitcoin (below 10 MB, 10–100 MB, above 100 MB) and by a median split on daily transaction count for Ethereum. Third, a critical temperature threshold is selected by grid search over admissible temperature quantiles, choosing the split that maximizes the distributional separation in fee outcomes.

The structural break analysis uses a batching variable as the independent variable in the temperature regression. For Bitcoin, batching is measured as the average number of outputs per transaction (avg_outputs_per_tx), capturing how many payment operations are bundled into each on-chain transaction. For Ethereum, batching is measured as the ratio of transfer count to transaction count (transfers per tx), capturing the analogous bundling of value transfers per transaction. Higher batching implies more efficient use of blockspace per fee-paying transaction.

Structural break analysis then tests whether the batching–temperature relation changes at economically meaningful protocol dates. On the Bitcoin side, the primary break date is 1 February 2023, corresponding to the ordinals shock in the frozen design. On the Ethereum side, the primary break date is 5 August 2021, corresponding to EIP-1559, while 15 September 2022 (the merge) and 12 April 2023 (Shanghai, also known as Shapella) are treated as secondary robustness windows. This event study logic is consistent with Choi [33], who links the merge to faster block times, higher throughput, and increased informed trading, and with Liu et al. [34], who show post-merge improvements in Ethereum liquidity and modest improvements in efficiency relative to Bitcoin. Appendix C documents the event chronology and the rationale for each break date.

### 2.9. Computational Environment and Replication Footprint

This study is an empirical diagnostic pipeline, not a deployed blockchain protocol, trading system, or simulation environment, so no on-chain overhead, gas cost, or validator burden is introduced. The replication package is implemented in Python 3.11+ (Python Software Foundation, Wilmington, DE, USA) with the following open-source libraries: NumPy, pandas, SciPy, statsmodels, matplotlib (NumFOCUS, Austin, TX, USA), and arch (Kevin Sheppard, University of Oxford, Oxford, UK). Variable construction and aggregation scale linearly in the number of observations; the Maxwell block scales as O(R·B·N), where R = 4 relations, B is the number of quantile bins, and N is the number of observations. The full pipeline runs in approximately 67 s with peak memory use of 384 MB on a standard desktop. The compressed archive is 4.93 MiB (9.84 MiB uncompressed) and contains all code, data, intermediate results, figures, tables, and documentation.

## 3. Results

### 3.1. Descriptive Temperature Landscape

The descriptive evidence already suggests that the two chains do not exhibit the same empirical regularities with equal clarity. Bitcoin’s fee-based temperature proxy is markedly more bounded: its daily mean is −7.44, its standard deviation is 1.06, and even its upper-tail realizations remain within a comparatively narrow range. Ethereum’s temperature operates at a higher and wider range, with a daily mean of 18.43, a standard deviation of 2.57, and realizations entirely in positive territory. The difference in level reflects the gwei denomination of Ethereum fees, but the dispersion contrast is substantive: Bitcoin’s temperature series behaves like a tighter congestion-and-fee state, whereas Ethereum’s series tracks a much broader and more persistent set of pricing conditions.

**Figure 1:**
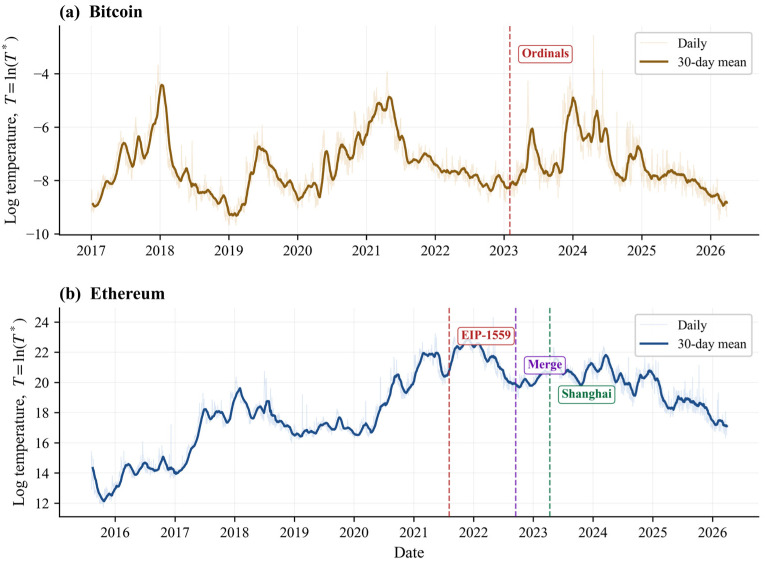
Daily log-temperature series for Bitcoin (**a**) and Ethereum (**b**) with protocol-event markers. Note. The difference in vertical scale reflects the gwei denomination of Ethereum fees (see Section 2.3). Bitcoin T ranges from approximately −10 to −3; Ethereum T ranges from approximately 12 to 24. Standard deviations are 1.06 (Bitcoin) and 2.57 (Ethereum). The gwei-to-ETH conversion does not affect the diagnostic results, as it adds only a constant to the log series.

Figure 1 reinforces this contrast visually. The Bitcoin series oscillates within a narrower band, with clear but ultimately contained surges around major fee episodes. Ethereum exhibits longer swings, higher peaks, and more extended high-temperature plateaus. This descriptive pattern is consistent with Li et al. [18], who show that a temperature-like observable can track meaningful liquidity variation in a centralized Bitcoin limit order book setting. The present evidence extends that intuition from exchange microstructure to public blockspace markets, but it does so in a way that already hints at chain asymmetry.

### 3.2. Temperature as a State Variable

The state variable diagnostics confirm that Bitcoin is the clearer empirical realization of a bounded temperature process (Table 3). On the daily panel, Bitcoin rejects the unit root null with an ADF *p*-value of 0.01, while the KPSS statistic does not reject stationarity at conventional levels (*p* = 0.10). Its estimated AR(1) coefficient is 0.97, implying a half-life of approximately 20.69 days. This is highly persistent, but the persistence is that of a bounded process, not of indefinite drift.

**Figure 2:**
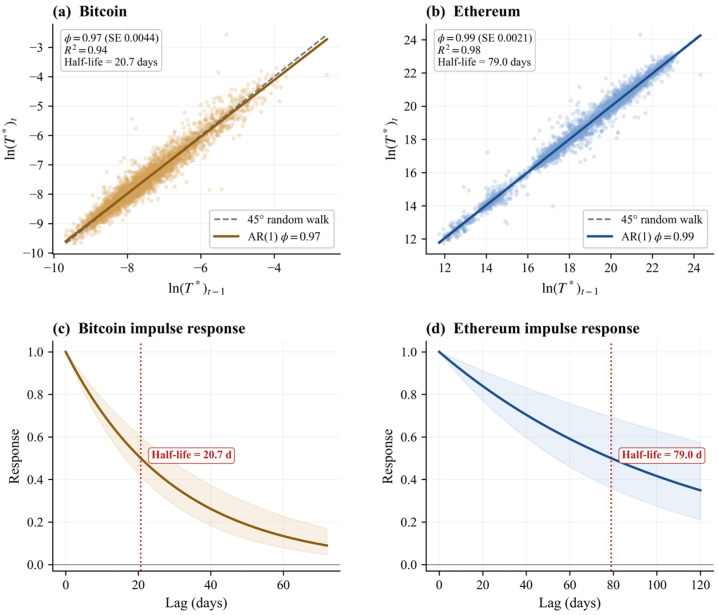
AR(1) mean reversion diagnostics for Bitcoin (**a**,**c**) and Ethereum (**b**,**d**). Note. Panels (**a**,**b**) plot ln(T*) against its one-day lag, with each circle representing one daily observation. Solid lines show the AR(1) fit; dashed grey lines indicate the 45° random-walk benchmark (in panel (**b**), the dashed line is largely obscured by the AR(1) fit because Ethereum’s φ = 0.99 is near unity). Panels (**c**,**d**) show the implied impulse response with 95% bootstrap confidence bands. Half-life = ln(0.5)/ln(phi).

Ethereum is materially weaker. Its ADF *p*-value is 0.19, so the unit root null is not rejected, while KPSS strongly rejects stationarity (*p* = 0.01). Its AR(1) coefficient of 0.99 implies a much longer half-life of 79.04 days. Ethereum temperature is highly persistent, with an AR(1) coefficient below unity but insufficient formal evidence to reject a unit root. Figure 2 illustrates this contrast: Bitcoin’s AR(1) fit departs visibly from the 45° random-walk benchmark, while Ethereum’s fit nearly overlaps it.

Bitcoin provides the stronger case. Ethereum remains informative, but as a near-unit root process, its mean reversion, if present, is too slow for the sample to confirm statistically.

### 3.3. Maxwell Relations Strengthen with Aggregation

**Figure 3:**
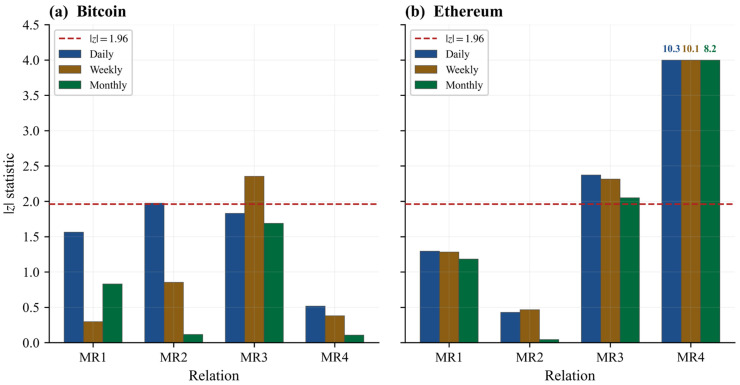
Maxwell-type z-statistics by chain and sampling frequency. Note. Bars below the dashed line (|z| = 1.96) indicate non-rejection of the cross-partial equality at the 5% level. MR4 values for Ethereum exceed the y-axis cap and are annotated numerically. All frequencies use 10 quantile bins. Under 6 bins for weekly and monthly panels, MR3 passes on both chains (see Section 3.7).

The Maxwell block asks whether the proposed T, S, P, V system behaves like an internally coherent state space rather than as a loose collection of correlated observables. The main specification uses 10 quantile bins at all frequencies (Table 4 and Figure 3; see Appendix A for the formal test construction).

For Bitcoin, the daily panel passes three of four Maxwell-type restrictions, with MR2 failing marginally (*p* = 0.05). At the weekly frequency, Bitcoin again passes three of four, with MR3 now the marginal failure (*p* = 0.02). At the monthly frequency, all four relations pass. The improvement at monthly frequency is also visible in the average magnitude of the deviations: mean absolute z-statistics fall from 1.47 on the daily panel to 0.97 weekly and 0.69 monthly. In other words, the conditional equalities become numerically tighter as the observation horizon lengthens, and Bitcoin achieves full integrability support at monthly frequency.

Ethereum passes two of four relations at all three frequencies. MR1 and MR2 are consistently supported; MR3 is marginal (z = 2.37 daily, 2.31 weekly, 2.05 monthly); and MR4 is the persistent failure, with z-statistics of 10.29 daily, 10.10 weekly, and 8.19 monthly. The MR4 deviation is not a borderline result but a large and stable rejection, indicating that one specific integrability condition does not hold in Ethereum’s state space. This is why Ethereum should be treated as a boundary case, not as a symmetric confirmation.

#### Theory-Driven Interpretation of Ethereum’s MR4 Failure

Ethereum’s persistent MR4 rejection is not a numerical anomaly. MR4 is the most demanding closure condition because it links the response of state complexity to pressure at fixed temperature, ${\left(\frac{\partial S}{\partial P}\right)}_{T}$, to the response of executed volume to temperature at fixed pressure, $-{\left(\frac{\partial V}{\partial T}\right)}_{P}$. This relation requires S, P, T, and V to be measured within the same effective information channel. That requirement is more plausible in Bitcoin’s public fee queue than in Ethereum’s layered transaction environment.

Three Ethereum-specific mechanisms can weaken this channel closure. First, MEV and priority-ordering markets create off-chain ordering channels, so the public pressure proxy does not necessarily capture the full pressure field faced by validators, builders, and searchers [35]. Second, EIP-1559 splits the user-paid fee into a burned base fee and a priority tip, so the monetary cost borne by users is no longer identical to validator revenue [36,37]. Third, after the merge, Ethereum validators serve simultaneously as stakeholders, introducing validator–staker coupling absent from Bitcoin’s miner fee architecture; Saleh [17] shows that validator incentives differ structurally when consensus participants are also asset holders, and post-merge evidence documents associated changes in trading and liquidity dynamics [33,34].

The Carnot and Maxwell results are therefore not contradictory. Carnot compliance is a one-dimensional efficiency-ratio constraint based on hot/cold temperature gradients; Maxwell consistency is a two-dimensional local-integrability condition involving paired cross-partial derivatives. A system can satisfy a broad efficiency bound while failing full state space closure. Ethereum is such a case: its temperature gradient is economically meaningful, but its full (T, S, P, V) state space is not fully integrable.

This interpretation is a theory-generating boundary condition hypothesis, not a fully identified causal mechanism. It is generated by the empirical pattern and requires independent validation with richer Ethereum-specific data on gas use, builder/relay routing, MEV extraction, and fee decomposition. The MR4 failure nevertheless identifies where the restricted thermodynamic mapping begins to break.

The pre-/post-EIP-1559 evidence gives the interpretation a testable form. If EIP-1559 created a split monetary channel by separating burned base fees from validator tips, the post-EIP period should not restore MR4 closure. The subperiod diagnostics are consistent with this weaker prediction: MR4 rejects both before and after EIP-1559, with z = 10.38 pre-EIP and z = 7.52 post-EIP under the 10-bin daily specification. The stronger claim (that rejection must become numerically larger after EIP-1559) is not supported and is not made here. The evidence supports the more conservative boundary claim: EIP-1559 does not repair MR4 integrability, and the post-EIP state space shows broader derivative instability.

### 3.4. Carnot-Style Constraints Are Broadly Satisfied

For Bitcoin, the implied Carnot-style bound is 0.89, whereas the mean realized fee extraction efficiency is only 0.05. Out of 1968 testable daily observations, only 38 violate the bound, yielding a daily compliance rate of 98.07%. The monthly heat–engine relation is also extremely tight, with a Pearson correlation of 0.979 between monthly fee revenue and the corresponding temperature-gap variable.

**Figure 4:**
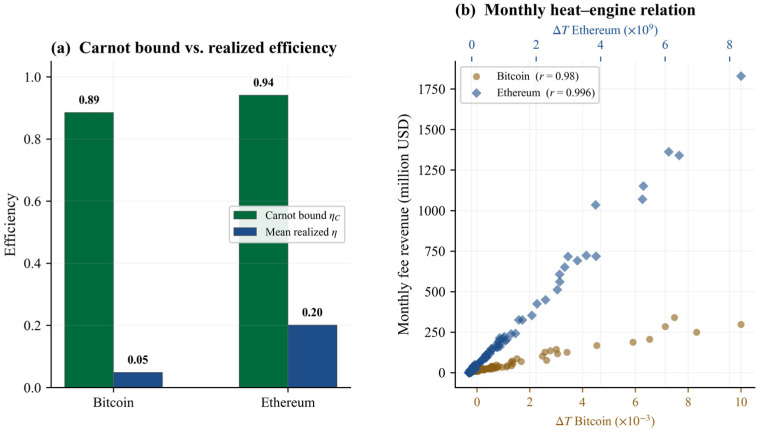
Carnot-style efficiency diagnostics for Bitcoin and Ethereum. Note. Panel (**a**) compares the implied Carnot bound ($\eta_{C}\;=\;1\;-\;\frac{T_{cold}}{T_{hot}}$) with mean realized fee extraction efficiency. Panel (**b**) plots monthly fee revenue against the temperature gap ΔT = $T_{month}$ − $T_{cold}$. Pearson correlations are reported in the legend.

Ethereum delivers similarly strong numerical support. Its implied Carnot bound is 0.94, while mean realized efficiency is 0.20. Out of 2088 testable daily observations, only 47 violate the bound, so daily compliance reaches 97.75%. The monthly heat–engine relation is even tighter than in Bitcoin, with r = 0.996. Figure 4 illustrates the bound efficiency gap and the monthly heat–engine relation.

Table 5 reports the Carnot diagnostics for both chains. Alternative regime definitions are reported in Appendix B and Table S2.

### 3.5. Temperature Regimes Are Nonlinear

The regime results show that temperature is not merely an average-level descriptor; it reorganizes across economically meaningful market states. In Bitcoin, the bull-versus-bear split is large and economically clear. The mean temperature is −6.91 in bull markets and −8.11 in bear markets, with a Kolmogorov–Smirnov statistic of 0.52 and a large standardized separation (d = 1.35). Bitcoin’s fee-based temperature is systematically higher in bull conditions. Figure S1 shows the full distributional separation.

Congestion regimes reinforce the same point. In Bitcoin, low-, medium-, and high-pressure states have mean temperatures of −8.02, −7.01, and −6.22, respectively, and the Kruskal–Wallis statistic is 1234.19. The ordering is monotonic and the pairwise separations are economically large, especially between low and high congestion (d = −2.52). That monotonic structure is exactly what a congestion-sensitive temperature proxy should produce.

Ethereum follows the same directional logic, but in a less fully integrated system. Bull and bear markets differ in the expected direction, with means of 18.97 and 18.38, although the effect size is smaller (d = 0.26). Congestion separation, however, is very strong: low- and high-pressure states have mean temperatures of 16.60 and 20.27, and the Kruskal–Wallis statistic reaches 2120.61. Ethereum does carry substantial nonlinear temperature structure, even if that structure coexists with weaker Maxwell support.

The critical threshold exercise sharpens the nonlinear interpretation. The estimated threshold is $T_{c}$ = −5.41 for Bitcoin and $T_{c}$ = 15.96 for Ethereum. Above those cut points, the fee outcome distribution changes sharply. The implication is that blockspace pricing is better understood as a threshold-sensitive regime process than as a single global linear fee schedule. Table 6 (Panel A) summarizes the regime thresholds for both chains.

Note. Panel A: Bull/Bear defined by price relative to 200-day moving average. Congestion tiers defined by fixed mempool size thresholds for Bitcoin (below 10 MB, 10–100 MB, above 100 MB) and by median split on daily transaction count for Ethereum (two tiers only). $T_{c}$ is the critical temperature threshold estimated by grid search. Panel B: Chow F tests the null of parameter stability in the log(T*) ~ batching regression at the indicated break date. All break tests use the log(T*) ~ batching specification. All breaks significant at the 1% level.

### 3.6. Protocol Events Restructure the Temperature Relation

The structural break results show that major protocol events do not merely shift activity levels; they reorganize the batching–temperature relation itself. The goal is not to claim “phase transitions” in a literal physical sense, but to show that protocol redesign and protocol shocks change the mapping between information batching and fee-based temperature.

For Bitcoin, Bertucci [29] shows that the first ordinals wave increased overall fees and miner revenue. For Ethereum, Jain et al. [28] show that blockchain fees increase nonlinearly with congestion and report a fee reduction around the merge. Choi [33] documents shorter block times, higher transaction throughput, and increased informed trading post-merge, while Liu et al. [34] find improved Ether liquidity and slight efficiency gains after the transition to proof-of-stake. The selection rationale for each break date is detailed in Appendix C.

In Bitcoin, the primary ordinals specification uses the 1 February 2023 break date in the log-temperature model. The Chow statistic is 19.83 and the post-break slope falls by 34.31%. Nearby robustness windows also remain significant: 1 December 2022 yields F = 26.75 with a −71.23% slope change, 1 January 2023 yields F = 19.19 with −51.89%, 1 March 2023 yields F = 23.46 with −23.34%, and 1 April 2023 yields F = 26.97 with −14.35%. The magnitude varies, but the break evidence is stable.

**Figure 5:**
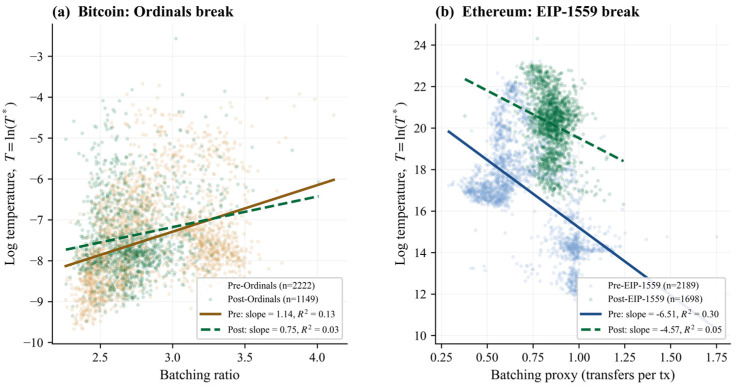
Structural breaks in the temperature–batching relation for Bitcoin (**a**) and Ethereum (**b**). Note. Solid lines show pre-event OLS fits; dashed lines show post-event fits. Bitcoin break date: 1 February 2023 (ordinals). Ethereum break date: 5 August 2021 (EIP-1559). Slope and R^2^ values are reported in the legend.

Ethereum’s structural breaks are even larger. The primary EIP-1559 log-specification break on 5 August 2021 yields a Chow statistic of 2037.22 and a +29.77% slope change. Secondary protocol windows also produce strong breaks: the merge on 15 September 2022 yields F = 489.25 with a +103.29% slope change, and Shanghai on 12 April 2023 yields F = 320.58 with a +132.62% slope change. Ethereum’s batching–temperature relation is not just time-varying: it is highly sensitive to protocol architecture. Table 6 (Panel B) reports the break estimates, and Figure 5 displays the pre- and post-event regression fits for both chains.

### 3.7. Robustness

The robustness analysis examines three issues: Maxwell sensitivity to discretization, Carnot sensitivity to regime definition, and subperiod stability. The Maxwell tests are sensitive to sample size and binning because the estimator uses within-bin slopes to approximate local partial derivatives, creating a bias–variance tradeoff. Too few bins oversmooth the conditional derivative; too many bins reduce the number of observations per bin and increase slope noise. The tradeoff is sharpest for monthly data, where aggregation reduces market microstructure noise but also reduces the number of observations available for each conditional bin.

Table S1 therefore reports Maxwell pass matrices across 5, 6, 8, 10, 15, and 20 quantile bins. The qualitative findings are stable, but individual relations differ in their sensitivity to bin choice. MR1 and MR2 are the most robust across both chains. MR3 is the most bin-sensitive, especially under finer discretization. Ethereum’s MR4 rejection is the most stable negative result: it fails across all tested bin counts and frequencies, indicating that the rejection is not a discretization artifact. Bitcoin’s monthly result deserves careful interpretation. Under the main 10-bin specification, Bitcoin passes all four Maxwell relations at monthly frequency. Unlike the daily and weekly panels, where MR3 weakens under finer binning, Bitcoin’s monthly panel passes all tested bin specifications. The smaller monthly sample still warrants caution, so the appropriate conclusion is not that Bitcoin satisfies literal thermodynamic integrability but that it displays the strongest and most aggregation-sensitive evidence of approximate low-frequency integrability. Carnot diagnostics are more robust than Maxwell diagnostics. Daily compliance remains above 96% across every tested hot/cold regime definition on both chains. This is expected: the Carnot block tests a broad ratio-bound constraint, not local cross-partial equality. Subperiod diagnostics are weaker, especially for stationarity and Maxwell pass counts, reflecting both reduced sample size and genuine regime dependence. The subperiod evidence is therefore not a failure of the framework but a reminder that the evidence is strongest in the full-sample and low-frequency diagnostics and weaker in shorter architectural windows.

## 4. Discussion

### 4.1. Interpretation of the Evidence

The thermodynamic framework adds structure that standard economic analysis does not provide: the Carnot bound imposes a first principles efficiency limit, the Maxwell tests evaluate state space coherence, and the five-layer hierarchy provides a stronger evidentiary standard than any single regression. Standard fee market analysis can describe that fees rise with congestion; the thermodynamic mapping asks whether fees, congestion, throughput, and chain state jointly satisfy the internal consistency conditions of a state system.

Parrondo et al. [1] argue that information must be incorporated into thermodynamics as a physical entity, while Horowitz & Esposito [2] show that when systems exchange information, the entropy balance acquires an additional information-driven component. Seifert [3] extends this logic to fluctuating nonequilibrium systems.

The evidence is not uniform: Ethereum does not reproduce the Bitcoin pattern symmetrically, and the results are strongest where the framework implies broad constraints, especially the Carnot block, and weaker where the theory requires full state space integrability. Blockspace markets are not “thermodynamic” in a literal or universal sense, but they display reproducible empirical regularities under a Landauer-based temperature mapping.

Standard economic theory evaluates financial architectures through optimization, equilibrium, or risk allocation. Davydov et al. [38] compare banking and peer-to-peer lending risks using a Markowitz-style portfolio framework. Their central result is that, under fixed borrower risk assumptions, the P2P lending model minimizes average nonsystematic investor risk relative to a two-tier banking model. Their focus is therefore architectural risk allocation: how different institutional structures redistribute a given risk matrix across investors and intermediaries.

The present paper asks a different question. It does not optimize a portfolio, minimize variance, or derive an equilibrium allocation. It asks whether fee-based settlement systems generate a coherent thermodynamic state representation when transaction fees are interpreted as the monetary cost of irreversible information processing. In the Davydov et al. framework, the central object is a risk functional. In the present framework, the central object is a latent settlement potential whose derivatives define temperature-like and pressure-like variables. Thus, the two approaches are complementary. Conventional economic theory explains optimization and risk allocation; the Landauer-based framework tests state space coherence, integrability, and efficiency bounds in transparent information-processing markets.

### 4.2. Bitcoin as the Primary Empirical Case

Bitcoin is the stronger empirical case because its fee market most closely matches the canonical object that the Landauer-based theory requires: a congestion-priced queue in which the cost of prioritized ledger inclusion is directly observable [13,14,15].

That structure helps explain why Bitcoin delivers the clearest boundedness evidence, the strongest gains from temporal aggregation, and the most interpretable break dynamics. Even when its daily data are noisy, Maxwell-type consistency reaches full support at monthly frequency, suggesting that the underlying regularity is present and becomes clearer once high-frequency noise is averaged out.

Bitcoin’s stationarity holds on the full sample but weakens in subperiods (Table S3); ADF fails to reject the unit root in pre-ordinals, post-ordinals, and most other subsample windows. This raises the possibility that full-sample mean reversion partly reflects level shifts between regimes rather than within-regime boundedness. The Carnot and structural break results, which do not depend on stationarity, are unaffected by this concern; the Maxwell and state variable interpretations should be read with this qualification in mind.

Biais et al. [16] argue that the fundamental value of a cryptocurrency depends on a stream of net transactional benefits, while relevant costs include fees paid to have transactions mined. When fees are economically central rather than peripheral, a fee-based temperature proxy is more likely to capture genuine structure. Bitcoin is not simply the chain with stronger statistics: it is the chain whose market organization is most closely aligned with the theory’s observable core.

### 4.3. Ethereum as a Boundary Condition Case

Ethereum matters because it is not a clean replication of Bitcoin. Bitcoin is the canonical case for the present design: a public congestion-priced queue in which user-paid fees visibly price scarce settlement capacity. Ethereum also prices settlement, but through a more layered architecture involving gas-based execution, smart contracts, private routing, MEV extraction, EIP-1559 fee burning, and proof-of-stake validator incentives.

The empirical pattern matches this architectural distinction. Ethereum satisfies the broad thermodynamic efficiency constraint: Carnot-style compliance exceeds 97%, and the monthly heat–engine relation is extremely high. The fee-based temperature gradient is therefore meaningful. However, Ethereum does not satisfy full Maxwell integrability: MR1 and MR2 pass in the full-sample main specification, MR3 is bin-sensitive, and MR4 fails persistently. The MR4 failure is informative because MR4 is the relation most exposed to unobserved pressure channels. It requires the state complexity response to pressure at fixed temperature to match the volume response to temperature at fixed pressure. That restriction is unlikely to hold when pressure is partly routed off-chain, when fee flows are split between burning and validator compensation, and when validators double as asset holders. Ethereum has all three features. This does not contradict the Carnot result. Carnot compliance is a one-dimensional efficiency bound; Maxwell integrability is a two-dimensional derivative closure requirement. Ethereum satisfies the first while failing the second. The interpretation is therefore architectural: the Landauer-based temperature mapping travels beyond Bitcoin, but full (T, S, P, V) state space closure weakens when the settlement system contains hidden ordering channels, burned fees, and validator–staker coupling. This interpretation is a boundary condition hypothesis, not a final causal explanation. If it is correct, Ethereum-specific data on base fee burns, priority tips, relay/builder routing, and MEV extraction should explain part of the MR4 deviation. Future work should test this using richer data, including gas use, relay concentration, and validator behavior. Ethereum does not invalidate the thermodynamic framework; it identifies where the minimal four-variable mapping ceases to be sufficient.

### 4.4. Contribution to Econophysics and Market Microstructure

This paper converts a recent theoretical proposal into a falsifiable empirical program, applying the standard urged by Yakovenko & Rosser [5] and Bouchaud [7] that econophysics should be disciplined by data. The results extend Li et al. [18] and Zhong et al. [19] from centralized order books to public settlement infrastructure and connect to the information-intensive trading environments studied by Chordia et al. [24] and Brogaard et al. [27]. On the practical side, the temperature framework offers a diagnostic lens for protocol designers, and the Carnot bound provides a benchmark against which fee extraction efficiency can be monitored.

### 4.5. Limits and Future Research

#### Falsifiability

The framework is empirically falsifiable in the present domain. It would be substantially weakened if three conditions held simultaneously. First, the fee-based temperature proxy would show no boundedness, persistence structure, regime separation, or protocol-event sensitivity beyond what is mechanically implied by asset prices. Second, Carnot-style compliance would fail systematically or collapse under alternative hot/cold regime definitions. Third, Maxwell-type restrictions would fail across all relations, chains, frequencies, and binning choices, with no improvement under temporal aggregation. The evidence does not satisfy these falsification conditions. Carnot compliance is robust on both chains, Bitcoin’s Maxwell structure strengthens with aggregation, and protocol events reorganize the temperature relation. At the same time, Ethereum’s MR4 failure and weak stationarity evidence show that the framework is not universally confirmed. The result is therefore partial support with identifiable boundary conditions, not unrestricted validation.

This paper has clear limits. First, the proxy construction is chain-specific. Bitcoin and Ethereum do not share identical state architectures, transaction models, or congestion objects, so the S, P, and V mappings are analogous rather than symmetric. The results should be read as evidence on a family of mappings rather than on one universally fixed thermodynamic coordinate system.

Second, the design is observational, not causal. Even when protocol dates generate strong structural breaks, this paper does not identify a single causal channel with the precision of a laboratory intervention. It shows reorganizations in the batching–temperature relation around economically meaningful events, not a fully isolated mechanism.

Third, blockspace markets are special because they are transparent. Fees, waiting times, and transaction processing priority are unusually visible in Bitcoin-style systems [13,14]. That transparency makes this setting a strong first laboratory, but results may not transport automatically to conventional financial markets where information-processing costs are less directly observable.

Fourth, the paper does not test economic entropy in the fuller sense proposed by Bormashenko & Shendrik [8], nor does it validate the full Carathéodory program. It tests a narrower temperature-centered subset.

Future research should move in two directions. One is horizontal extension across other fee infrastructures: rollups, layer-2 settlement systems, stablecoin transfer rails, and decentralized exchange sequencing layers. The other is vertical refinement: better chain-comparable state variables, richer regime definitions, and direct attempts to operationalize economic entropy rather than stopping at temperature alone.

## 5. Conclusions

This paper asked whether blockspace markets exhibit coherent empirical regularities when economic temperature is operationalized as a Landauer-based cost of irreversibly processed information [8]. The answer is affirmative but qualified: the evidence is partial, frequency-dependent, and chain-specific. Carnot-style constraints are the strongest result, with compliance above 97% on both chains. Maxwell-type integrability reaches full support for Bitcoin at monthly frequency but remains incomplete for Ethereum. Structural breaks confirm that protocol events reorganize the temperature relation rather than merely shifting activity levels.

These findings go beyond what standard fee market economics can deliver: a first principles efficiency bound, a state space coherence test, and a five-layer diagnostic hierarchy that no single regression or supply-demand model provides. Theoretically, the Landauer-based framework can be translated from a conceptual statement into a falsifiable empirical design, consistent with the thermodynamics-of-information perspective in which information carries measurable physical consequences [1]. Empirically, the paper offers a replication-ready template for studying fee-based information infrastructures beyond the present setting.

## Figures and Tables

**Table 1:** Thermodynamic notation: symbols, roles, and empirical status.

Symbol	Name	Definition/Formula	Empirical Status
$E^{act}$	Realized information-processing cost	Monetary cost of one irreversibly processed bit	Observed
$T^{*}$	Level economic temperature	$E^{act}/ln2$	Constructed
T	Log economic temperature	$ln(T^{*})$	Constructed
S	State complexity coordinate	Entropy-side coordinate (chain-specific proxy)	Proxy
P	Demand-pressure coordinate	Pressure-side coordinate (chain-specific proxy)	Proxy
V	Settlement-volume coordinate	Volume-side coordinate (chain-specific proxy)	Observed/proxy
U(S,V)	Primitive settlement potential	dU=T dS−P dV	Latent
H(S,P)	Enthalpy-type potential	H=U+PV; dH=T dS+V dP	Implied
F(T,V)	Helmholtz-type potential	F=U−TS; dF=−S dT−P dV	Implied
G(T,P)	Gibbs-type potential	G=U−TS+PV; dG=−SdT+VdP	Implied
MR1–MR4	Maxwell-type restrictions	Cross-partial equalities from dU, dH, dF, dG	Tested (Section 3.3)

Note. Natural logarithms throughout. Observed: Read directly from chain data; Constructed: computed from observables; Proxy: chain-specific stand-in (see Table 2); Latent: assumed scalar potential, not estimated; Implied: Legendre transform of U(S, V); Tested: integrability restriction evaluated in Section 3.3.

**Table 2:** Thermodynamic variable mapping for Bitcoin and Ethereum.

Construct	Symbol	Bitcoin	Ethereum	Interpretation
Temperature	T	$log\left(\frac{fee\_rate\cdot P_{BTC}}{8\cdot ln2\cdot 10^{8}}\right)$	$log\left(\frac{fee\_per\_tx\_gwei\cdot P_{ETH}}{8\cdot ln2}\right)$	Fee-based economic temperature
Entropy	S	log(utxo_count)	log(tx_count)	Chain state complexity
Pressure	P	log(mempool_size_bytes)	log(active_addresses)	Congestion/Demand pressure
Volume	V	avg_block_size_mb	log(block_count)	Executed blockspace volume

Note. All logarithms are natural. Bitcoin: fee_rate in satoshis per virtual byte; $P_{BTC}$ in USD per Bitcoin; constants 8 (bytes→bits) and $10^{8}$ (satoshis→BTC). Ethereum: fee_per_tx_gwei in gwei per transaction; $P_{ETH}$ in USD per ether. See Section 2.3 for the frozen Ethereum specification and the absorption of the gwei-to-ETH constant in derivative-based diagnostics. Bitcoin daily clean sample: 3371 observations, 1 January 2017–28 March 2026. Ethereum daily clean sample: 3887 observations, 8 August 2015–29 March 2026.

**Table 3:** Descriptive statistics and state variable diagnostics for the temperature proxy.

Chain	N	Mean	SD	Min	Max	ADF p	KPSS *p*	AR(1) phi	Half-Life (Days)
BTC	3371	−7.44	1.06	−9.68	−2.57	0.01	0.10	0.97	20.69
ETH	3887	18.43	2.57	11.73	24.31	0.19	0.01	0.99	79.04

Note. Statistics are for log-temperature *T* = ln(T*), where T* is the Landauer-based temperature proxy (see Section 2.3 for the chain-specific mappings). Absolute levels are not cross-chain comparable: Bitcoin is standardized as USD per bit, while Ethereum uses a frozen gwei-denominated effective settlement cost proxy. ADF tests the null of a unit root; KPSS tests the null of stationarity. AR(1) phi is the first-order autoregressive coefficient; half-life in days = ln(0.5)/ln(phi).

**Table 4:** Maxwell-type cross-partial restrictions by chain and sampling frequency.

Chain	Frequency	N	Passes	Failed	Mean |z|
BTC	Daily	3371	3/4	MR2	1.47
BTC	Weekly	483	3/4	MR3	0.97
BTC	Monthly	111	4/4	None	0.69
ETH	Daily	3887	2/4	MR3, MR4	3.60
ETH	Weekly	556	2/4	MR3, MR4	3.54
ETH	Monthly	128	2/4	MR3, MR4	2.87

Note. A pass indicates non-rejection of the cross-partial equality restriction at the 5% level (|z| < 1.96). All frequencies use 10 quantile bins. Mean |z| is the average absolute z-statistic across the four relations. MR3 is sensitive to binning specification; under 6 bins for weekly and monthly panels, MR3 passes on both chains at all frequencies (see Section 3.7).

**Table 5:** Carnot-style efficiency diagnostics.

Chain	Carnot Bound	Mean Efficiency	Testable Days	Violations	Compliance %	Heat-Engine r
BTC	0.89	0.05	1968	38	98.07	0.979
ETH	0.94	0.20	2088	47	97.75	0.996

Note. The Carnot-style bound is computed as $\eta_{C}\;=\;1\;-\;\frac{T_{cold}}{T_{hot}}$, where hot and cold regimes are defined by fixed mempool size thresholds for Bitcoin (cold < 10 MB, hot > 100 MB) and by a median split on daily fee per transaction for Ethereum (see Section 2.7). Mean efficiency is transaction fees as a share of total miner/validator revenue. Compliance is the share of testable days on which realized efficiency does not exceed the daily Carnot bound. Heat–engine r is the Pearson correlation between monthly fee revenue and Δ*T* = $T_{month}$− $T_{cold}$. Alternative regime definitions are reported in Appendix B and Table S2.

**Table 6:** Regime thresholds and structural break estimates.

**Panel A. Regime thresholds**						
**Chain**	**Bull Mean T**	**Bear Mean T**	**Low Congestion**	**Medium Congestion**	**High Congestion**	$T_{c}$
BTC	−6.91	−8.11	−8.02	−7.01	−6.22	−5.41
ETH	18.97	18.38	16.60	—	20.27	15.96
**Panel B. Structural breaks**						
**Chain**	**Event**		**Break Date**		**Chow F**	**Slope Change %**
BTC	Ordinals (primary)		1 February 2023		19.83	−34.31
ETH	EIP-1559 (primary)		5 August 2021		2037.22	+29.77
ETH	Merge (secondary)		15 September 2022		489.25	+103.29
ETH	Shanghai (secondary)		12 April 2023		320.58	+132.62

Note. Panel A: Bull/Bear defined by price relative to 200-day moving average. Congestion tiers defined by fixed mempool size thresholds for Bitcoin (below 10 MB, 10–100 MB, above 100 MB) and by median split on daily transaction count for Ethereum (two tiers only). $T_{c}$ is the critical temperature threshold estimated by grid search. Panel B: Chow F tests the null of parameter stability in the log(T*) ~ batching regression at the indicated break date. All break tests use the log(T*) ~ batching specification. All breaks significant at the 1% level.

## Data Availability

The replication package containing all code, data, and outputs is uploaded as Supplementary Materials alongside this submission.

## Supplementary Materials

The following supporting information can be downloaded at: https://www.mdpi.com/article/10.3390/e28050508/s1, Figure S1: Temperature distributions in bull and bear regimes for Bitcoin (a) and Ethereum (b); Table S1: Maxwell pass matrices under alternative bin specifications; Table S2: Carnot compliance and heat–engine correlations under alternative regime definitions; Table S3: Subperiod state variable and Maxwell diagnostics; Table S4: Sensitivity of main diagnostics to alternative temperature proxies.

## Appendix A. Maxwell Test Construction and Binning Rationale

Section 2.3 derives the Maxwell-type restrictions from the restricted scalar settlement potential U(S, V) and its Legendre transforms. This appendix documents how those restrictions are estimated empirically. The equalities are not assumed to hold exactly in noisy financial time series; they are used as approximate integrability diagnostics: if the mapped variables form a coherent state space, paired cross-partial derivatives should be statistically consistent after conditioning on comparable state regions. The four restrictions are:

$$MR1:{\left(\frac{\partial T}{\partial V}\right)}_{S}=-{\left(\frac{\partial P}{\partial S}\right)}_{V},$$

$$MR2:{\left(\frac{\partial T}{\partial P}\right)}_{S}={\left(\frac{\partial V}{\partial S}\right)}_{P},$$

$$MR3:{\left(\frac{\partial S}{\partial V}\right)}_{T}={\left(\frac{\partial P}{\partial T}\right)}_{V},$$

$$MR4:{\left(\frac{\partial S}{\partial P}\right)}_{T}=-{\left(\frac{\partial V}{\partial T}\right)}_{P}.$$

Each partial derivative is estimated by conditional binning. The conditioning variable is partitioned into quantile bins; within each bin, a local linear slope is estimated by OLS. The within-bin slopes are averaged to obtain the partial derivative estimate. The paired left-hand side and right-hand side estimates are compared using a z-statistic,

$$z=\frac{\left(\theta_{LHS}-\theta_{RHS}\right)}{\sqrt{{SE\left(\theta_{LHS}\right)}^{2}+{SE\left(\theta_{RHS}\right)}^{2}}}.$$

A relation is supported when |z| < 1.96. The main specification uses 10 quantile bins at all frequencies. Sensitivity to 5, 6, 8, 10, 15, and 20 bins is reported in Table S1.

## Appendix B. Carnot Formulation and Alternative Regime Definitions

The baseline Carnot bound is $\eta_{C}=1-\frac{T_{cold}}{T_{hot}}$, where $T_{cold}$ and $T_{hot}$ are the mean level temperatures $T^{*}$ in the cold and hot regimes, respectively (see Section 2.7 for regime definitions). For Bitcoin, regimes are defined from mempool size, an upstream congestion variable that is not a direct ingredient of $T^{*}$. For Ethereum, regimes are defined by a median split on daily fee per transaction, which is a component of $T^{*}$. This introduces a degree of mechanical dependence between the partition and the bounded variable. The dependence is mitigated by two factors: first, $T^{*}$ also depends on price, which varies independently of fee level; second, Table S2 shows that alternative partitions (terciles, quartiles, active address median split) yield compliance above 96% in all cases, confirming that the result is not an artifact of the baseline partition rule.

For robustness, the following alternative regime definitions are tested: tercile-based and quartile-based hot/cold partitions on the congestion proxy, and a median split on active addresses for Ethereum. Table S2 reports that daily compliance exceeds 96% across all variants on both chains.

## Appendix C. Event Chronology and Break Date Rationale

The principle of event selection is that protocol changes must plausibly alter the batching–temperature relation by changing settlement design, fee formation, or realized throughput.

For Bitcoin, the primary break date is 1 February 2023, capturing the onset of the ordinals inscription wave. Ordinals introduced nonpayment blockspace demand that competed with standard transactions for scarce block capacity, plausibly changing the mapping from batching to fee-based temperature [29].

For Ethereum, the primary break date is 5 August 2021, corresponding to EIP-1559, which changed the fee mechanism itself by introducing a dynamically adjusting base fee and variable-size blocks [36,37]. Secondary dates are 15 September 2022 (the merge, transitioning from PoW to PoS) and 12 April 2023 (Shanghai, enabling staking withdrawals). The hierarchy is analytical: EIP-1559 targets fee formation directly, while the merge and Shanghai are broader architectural changes that serve as robustness windows [33,34].

These dates are not claimed to exhaust all relevant changes in blockchain design. They are the most economically interpretable anchors for testing structural sensitivity in the batching–temperature relation.
