# Supplementary material for: Landauer-Based Economic Temperature in Blockspace Markets: Evidence from Bitcoin and Ethereum
Source: Entropy (Basel). 2026 May 1;28(5):508. doi: 10.3390/e28050508 (PMC13205438; doi:10.3390/e28050508)
Supplement: Supplementary file 1 [file entropy-28-00508-s001.zip › entropy-4271922-supplementary-main.pdf]

## Supplementary

**Table S1.** *Maxwell Pass Matrices Under Alternative Bin Specifications*

| Chain | Frequency | Bins | MR1 | MR2 | MR3 | MR4 | Pass |
|---|---|---|---|---|---|---|---|
| BTC | Daily | 5 | PASS | PASS | PASS | PASS | 4/4 |
| BTC | Daily | 6 | PASS | PASS | PASS | PASS | 4/4 |
| BTC | Daily | 8 | PASS | PASS | PASS | PASS | 4/4 |
| BTC | Daily | 10 | PASS | FAIL | PASS | PASS | 3/4 |
| BTC | Daily | 15 | PASS | PASS | FAIL | PASS | 3/4 |
| BTC | Daily | 20 | FAIL | PASS | FAIL | PASS | 2/4 |
| BTC | Weekly | 5 | PASS | PASS | FAIL | PASS | 3/4 |
| BTC | Weekly | 6 | PASS | PASS | PASS | PASS | 4/4 |
| BTC | Weekly | 8 | PASS | PASS | FAIL | PASS | 3/4 |
| BTC | Weekly | 10 | PASS | PASS | FAIL | PASS | 3/4 |
| BTC | Weekly | 15 | PASS | PASS | FAIL | PASS | 3/4 |
| BTC | Weekly | 20 | PASS | PASS | FAIL | PASS | 3/4 |
| BTC | Monthly | 5 | PASS | PASS | PASS | PASS | 4/4 |
| BTC | Monthly | 6 | PASS | PASS | PASS | PASS | 4/4 |
| BTC | Monthly | 8 | PASS | PASS | PASS | PASS | 4/4 |
| BTC | Monthly | 10 | PASS | PASS | PASS | PASS | 4/4 |
| BTC | Monthly | 15 | PASS | PASS | PASS | PASS | 4/4 |
| BTC | Monthly | 20 | PASS | PASS | PASS | PASS | 4/4 |
| ETH | Daily | 5 | PASS | PASS | PASS | FAIL | 3/4 |
| ETH | Daily | 6 | PASS | PASS | PASS | FAIL | 3/4 |
| ETH | Daily | 8 | PASS | PASS | FAIL | FAIL | 2/4 |
| ETH | Daily | 10 | PASS | PASS | FAIL | FAIL | 2/4 |
| ETH | Daily | 15 | PASS | PASS | FAIL | FAIL | 2/4 |
| ETH | Daily | 20 | FAIL | PASS | FAIL | FAIL | 1/4 |
| ETH | Weekly | 5 | PASS | PASS | PASS | FAIL | 3/4 |
| ETH | Weekly | 6 | PASS | PASS | PASS | FAIL | 3/4 |
| ETH | Weekly | 8 | PASS | PASS | FAIL | FAIL | 2/4 |
| ETH | Weekly | 10 | PASS | PASS | FAIL | FAIL | 2/4 |
| ETH | Weekly | 15 | PASS | PASS | FAIL | FAIL | 2/4 |
| ETH | Weekly | 20 | FAIL | PASS | FAIL | FAIL | 1/4 |
| ETH | Monthly | 5 | PASS | PASS | PASS | FAIL | 3/4 |
| ETH | Monthly | 6 | PASS | PASS | PASS | FAIL | 3/4 |
| ETH | Monthly | 8 | PASS | PASS | PASS | FAIL | 3/4 |
| ETH | Monthly | 10 | PASS | PASS | FAIL | FAIL | 2/4 |
| ETH | Monthly | 15 | FAIL | PASS | PASS | FAIL | 2/4 |
| ETH | Monthly | 20 | FAIL | PASS | FAIL | FAIL | 1/4 |

***Note.*** *PASS indicates non-rejection of the cross-partial equality at the 5% level (|z| < 1.96). FAIL entries are bolded. The main specification (10 bins at all frequencies) is reported in Table 4. MR1 and MR2 pass consistently across nearly all specifications on both chains. MR3 is the most sensitive relation, passing at 5-6 bins but failing at 8+ bins in most cases. MR4 fails consistently for Ethereum regardless of bin count. Bitcoin monthly achieves 4/4 across all bin specifications tested.*

**Table S2.** Carnot Compliance and Heat-Engine Correlations Under Alternative Regime Definitions

| Chain | Partition | η_C | Compliance % | Testable days | Violations | Heat-engine r |
|---|---|---|---|---|---|---|
| BTC | Baseline (10/100 MB) | 0.89 | 98.07 | 1,968 | 38 | 0.979 |
| BTC | Terciles (mempool) | 0.87 | 98.27 | 2,259 | 39 | 0.979 |
| BTC | Quartiles (mempool) | 0.90 | 98.30 | 2,355 | 40 | 0.979 |
| BTC | Median split (mempool) | 0.81 | 98.16 | 2,010 | 37 | 0.979 |
| ETH | Baseline (median fee) | 0.94 | 97.75 | 2,088 | 47 | 0.996 |
| ETH | Terciles (fee) | 0.97 | 98.84 | 2,232 | 26 | 0.996 |
| ETH | Quartiles (fee) | 0.97 | 98.50 | 2,199 | 33 | 0.996 |
| ETH | Median (active addresses) | 0.88 | 96.09 | 1,792 | 70 | 0.996 |

***Note.*** *Baseline regime definitions match Section 2.7: Bitcoin uses fixed mempool-size thresholds (cold < 10 MB, hot > 100 MB); Ethereum uses a median split on daily fee per transaction. Alternative partitions vary the quantile rule on the same congestion proxy. Compliance is the share of testable days on which realized efficiency does not exceed the daily Carnot bound. Heat-engine r is the Pearson correlation between monthly fee revenue and ΔT = T_month − T_cold. All variants show compliance above 96% and heat-engine r above 0.98, confirming that the Carnot result is not sensitive to the regime partition rule.*

**Table S3.** *Subperiod State-Variable and Maxwell Diagnostics*

| Chain | Period | N | ADF p | KPSS p | AR(1) φ | Half-life | Maxwell |
|---|---|---|---|---|---|---|---|
| BTC | Full sample | 3,370 | 0.01 | 0.10 | 0.97 | 20.6 | 3/4 |
| BTC | Pre-Ordinals | 2,222 | 0.09 | 0.04 | 0.98 | 32.3 | 2/4 |
| BTC | Post-Ordinals | 1,148 | 0.15 | 0.01 | 0.94 | 11.2 | 1/4 |
| ETH | Full sample | 3,886 | 0.19 | 0.01 | 0.99 | 79.3 | 2/4 |
| ETH | Pre-EIP-1559 | 2,189 | 0.68 | 0.01 | 0.99 | 86.4 | 1/4 |
| ETH | Post-EIP-1559 | 1,697 | 0.77 | 0.01 | 0.97 | 22.9 | 1/4 |
| ETH | Pre-Merge | 2,595 | 0.45 | 0.01 | 0.99 | 106.7 | 2/4 |
| ETH | Post-Merge | 1,291 | 0.81 | 0.01 | 0.96 | 16.4 | 1/4 |

***Note.*** *ADF tests the null of a unit root; KPSS tests the null of stationarity. Half-life in days = ln(0.5)/ln(φ). Maxwell column reports daily pass count out of 4 relations using 10 quantile bins. Bitcoin stationarity holds on the full sample (ADF rejects, KPSS does not reject) but weakens in subperiods. Ethereum fails to reject the*

*unit root in all subperiods. Maxwell support also weakens in subsamples, consistent with reduced statistical power from smaller samples. These results are discussed in Section 3.7.*

**Figure S1.** Temperature distributions in bull and bear regimes for Bitcoin (a) and Ethereum (b).

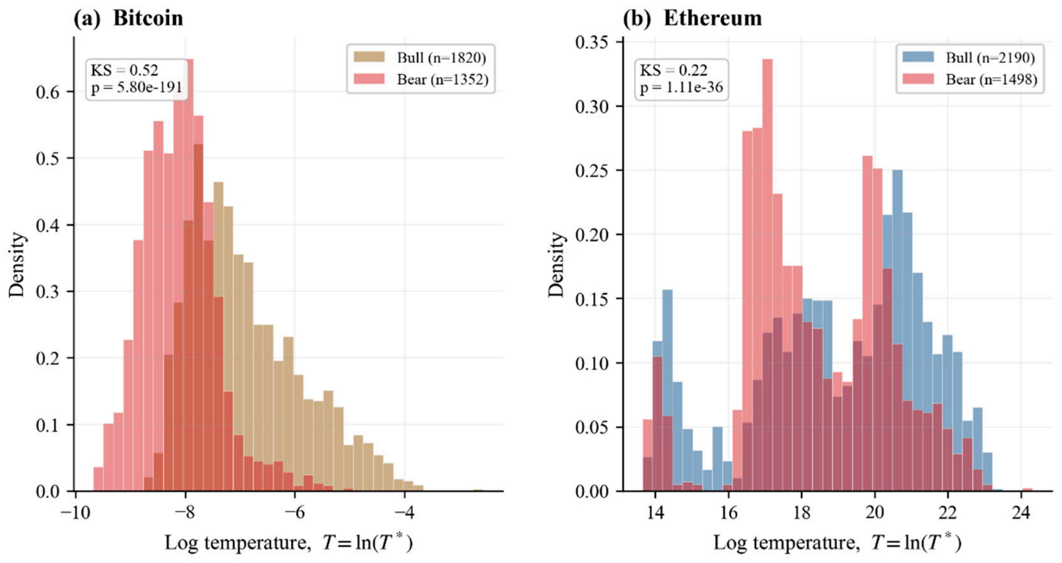


***Note.*** *Bull/bear classification based on price relative to 200-day moving average. KS = Kolmogorov-Smirnov two-sample statistic. Bitcoin shows clear distributional separation; Ethereum shows weaker separation consistent with the smaller effect size reported in Section 3.5.*

***Table S4****. Sensitivity of Main Diagnostics to Alternative Temperature Proxies.*

| Chain | Proxy | Unit | State-variable (ADF p) | Maxwell daily | Maxwell weekly | Maxwell monthly | Carnot compliance % | Main conclusion |
|---|---|---|---|---|---|---|---|---|
| BTC | Median sat/vB x BTC price | USD/bit | 0.01 | 3/4 | 3/4 | 4/4 | 98.07 | Canonical (matches main text) |
| BTC | mm.space avgfee_50 x BTC price | USD/bit | 0.32 | 2/4 | 3/4 | 2/4 | 99.66 | Consistent with canonical |
| BTC | Total fees / total vbytes | USD/bit | <0.01 | 4/4 | 3/4 | 4/4 | 97.15 | Consistent with canonical |
| ETH | Fee per tx x ETH price | gwei-USD/bit | 0.19 | 2/4 | 2/4 | 2/4 | 97.75 | Canonical (matches main text) |
| ETH | Total fees / total gas used | USD/gas | 0.20 | 2/4 | 2/4 | 2/4 | 97.89 | Consistent with canonical |

*Note. All proxies are computed at daily frequency. "Canonical" denotes the specification used in the main text (see Section 2.3). Maxwell pass counts use 10 quantile bins at each frequency. ADF p reports the unit-root test on the log-transformed proxy. Carnot compliance is the share of testable days on which realized efficiency does not exceed the daily Carnot bound. Bitcoin proxies: sat/vB = satoshis per virtual byte; mm.space avgfee_50 = Mempool.space 50th-percentile average fee. Ethereum proxies: gwei-USD/bit is the frozen fee-per-transaction specification; USD/gas is the gas-normalized native-unit alternative requested in the reviewer comments.*
