# Supplementary figures and images for: Landauer-Based Economic Temperature in Blockspace Markets: Evidence from Bitcoin and Ethereum
Source: Entropy (Basel). 2026 May 1;28(5):508. doi: 10.3390/e28050508 (PMC13205438; doi:10.3390/e28050508)

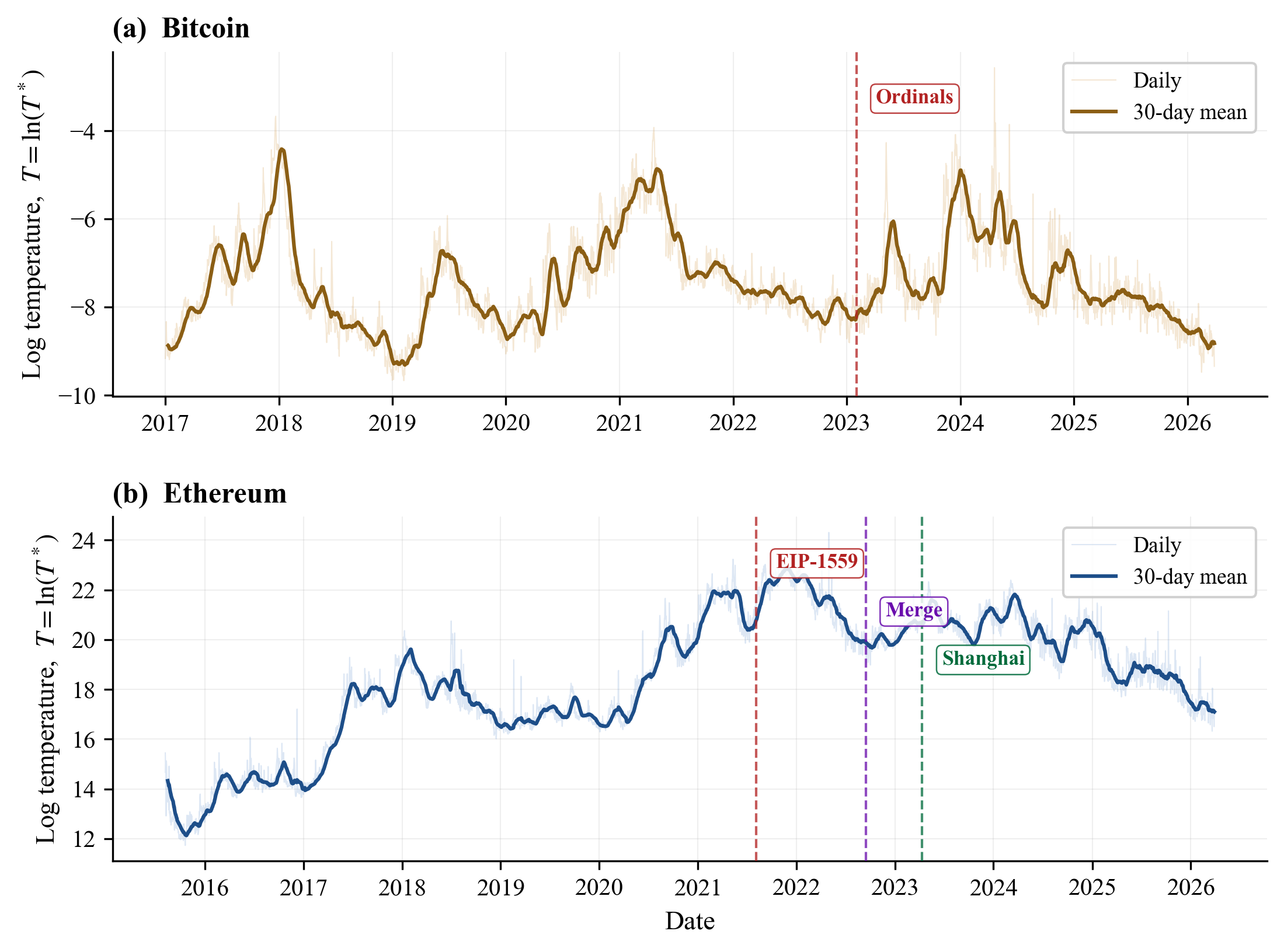

Supplement: Supplementary file 1 [file entropy-28-00508-s001.zip › entropy_submission_entropy_optimized/figures/fig1_temperature_timeseries.png]

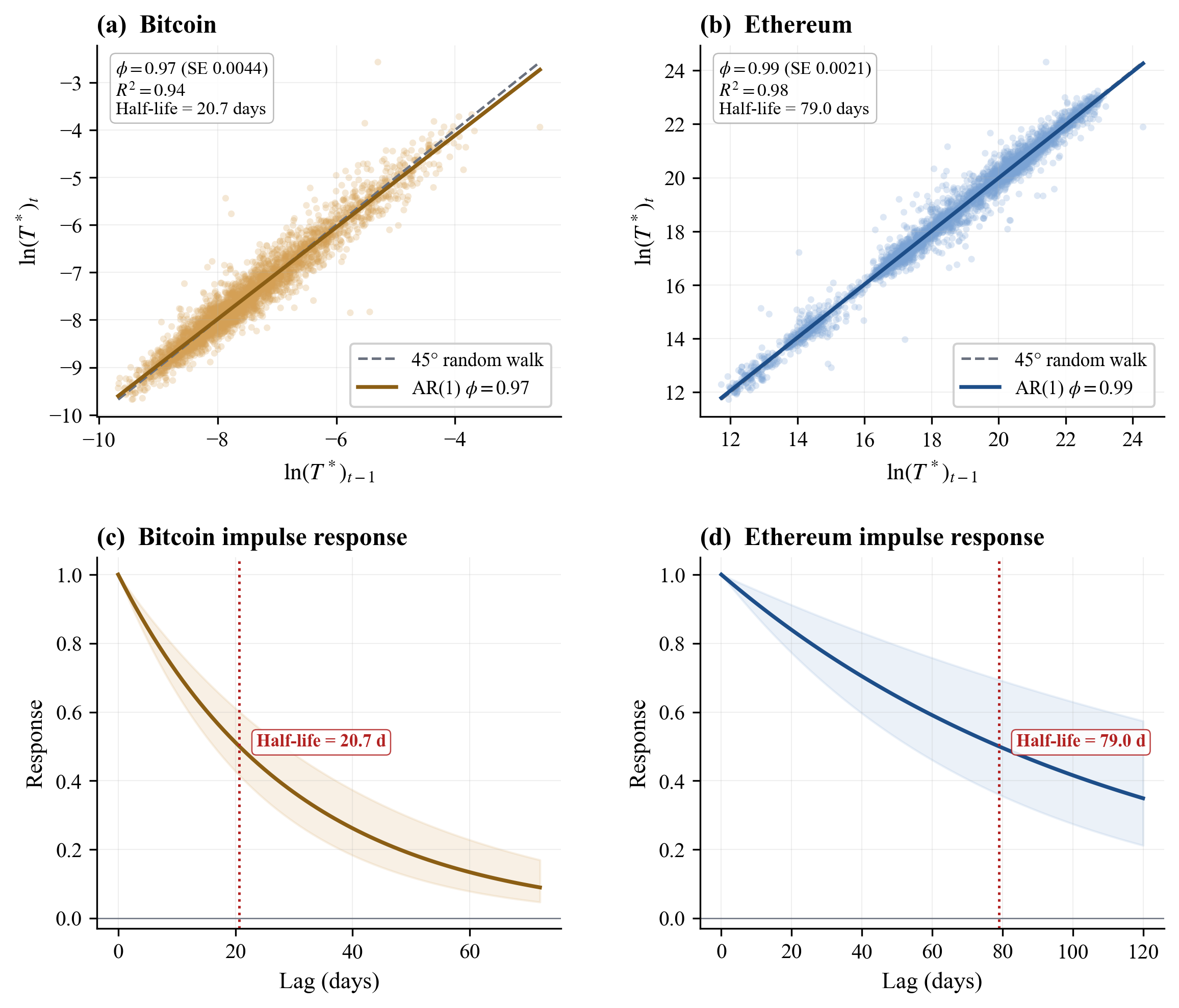

Supplement: Supplementary file 1 [file entropy-28-00508-s001.zip › entropy_submission_entropy_optimized/figures/fig2_mean_reversion.png]

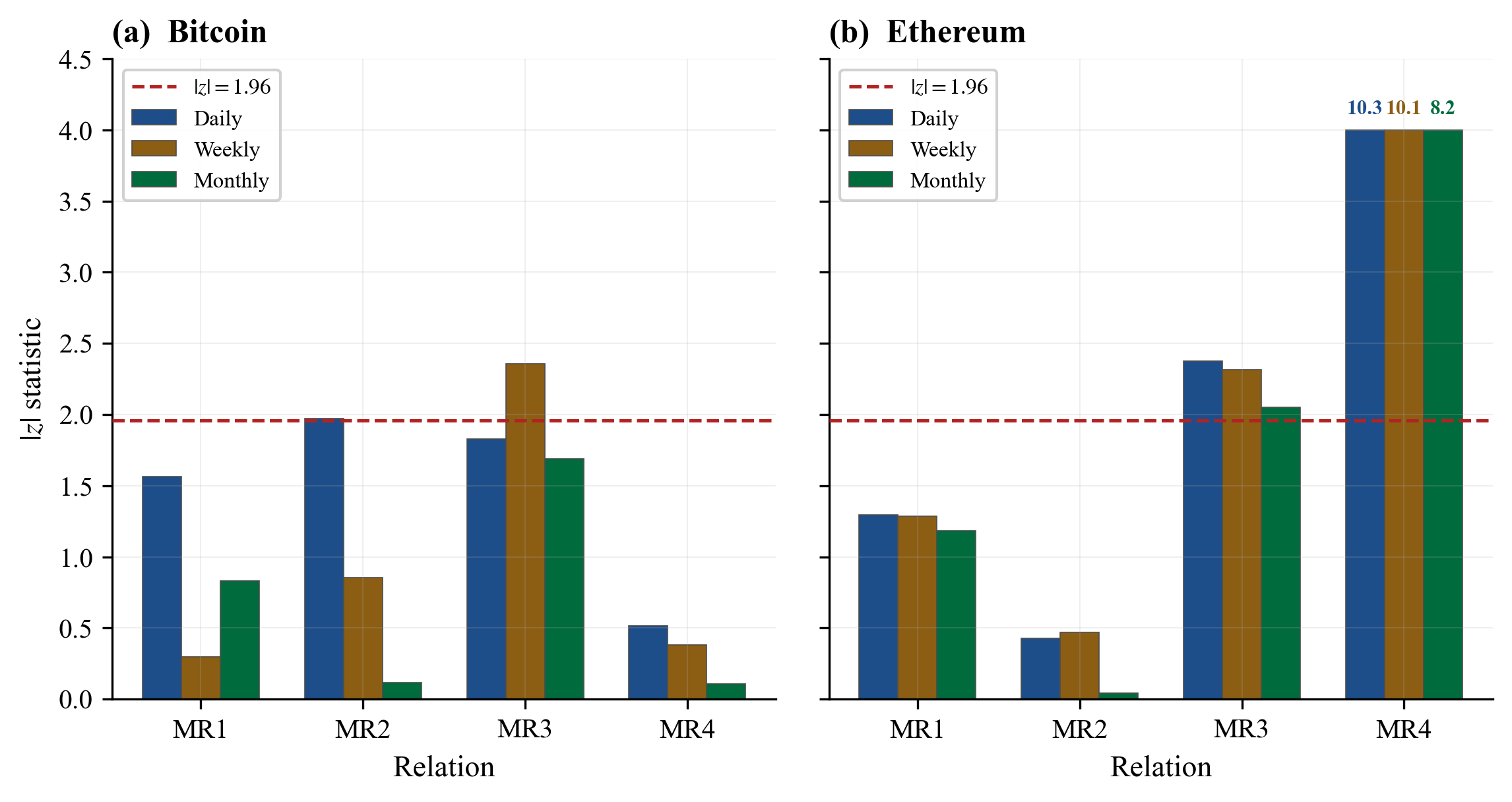

Supplement: Supplementary file 1 [file entropy-28-00508-s001.zip › entropy_submission_entropy_optimized/figures/fig3_maxwell_relations.png]

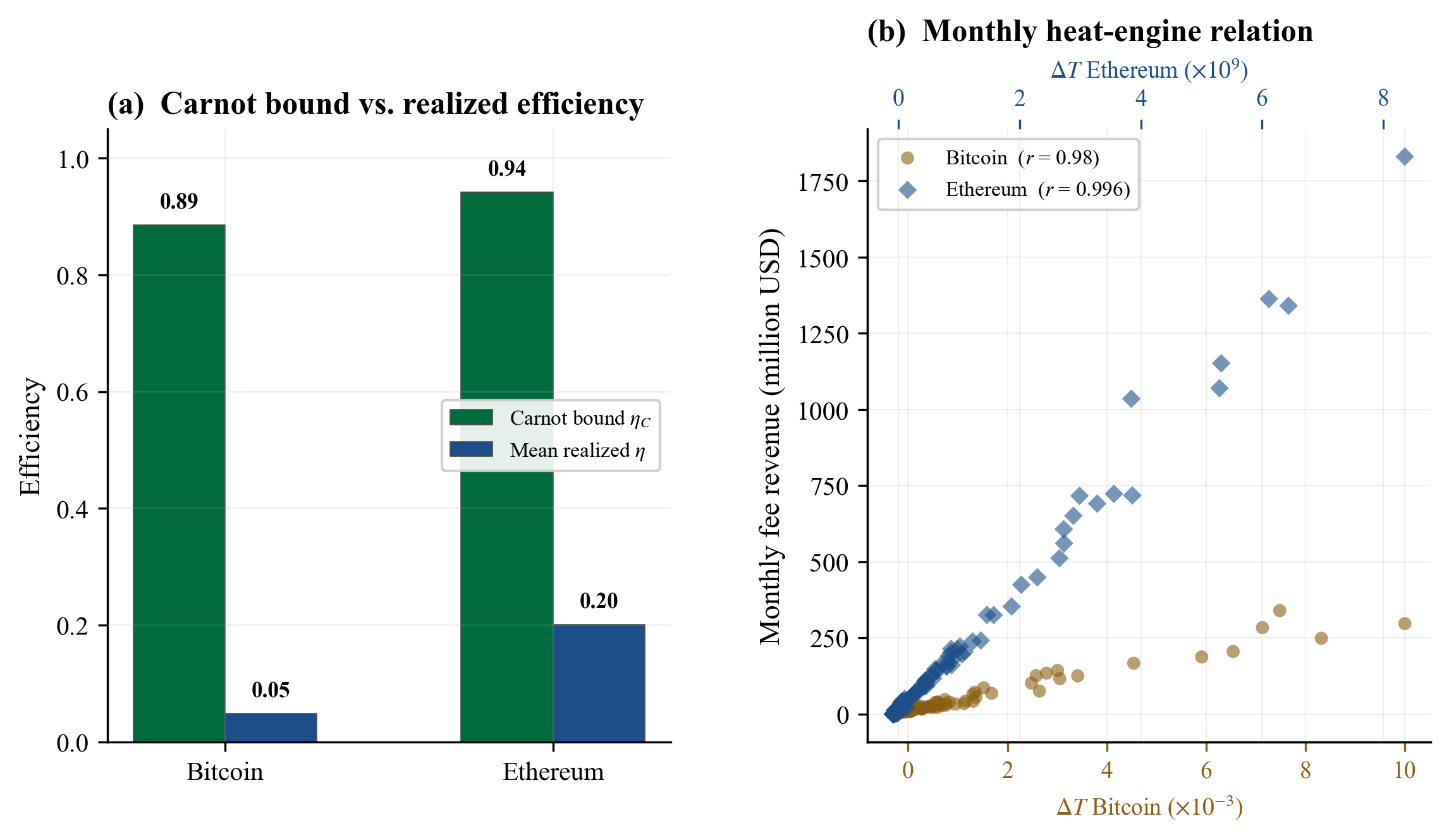

Supplement: Supplementary file 1 [file entropy-28-00508-s001.zip › entropy_submission_entropy_optimized/figures/fig4_carnot_support.png]

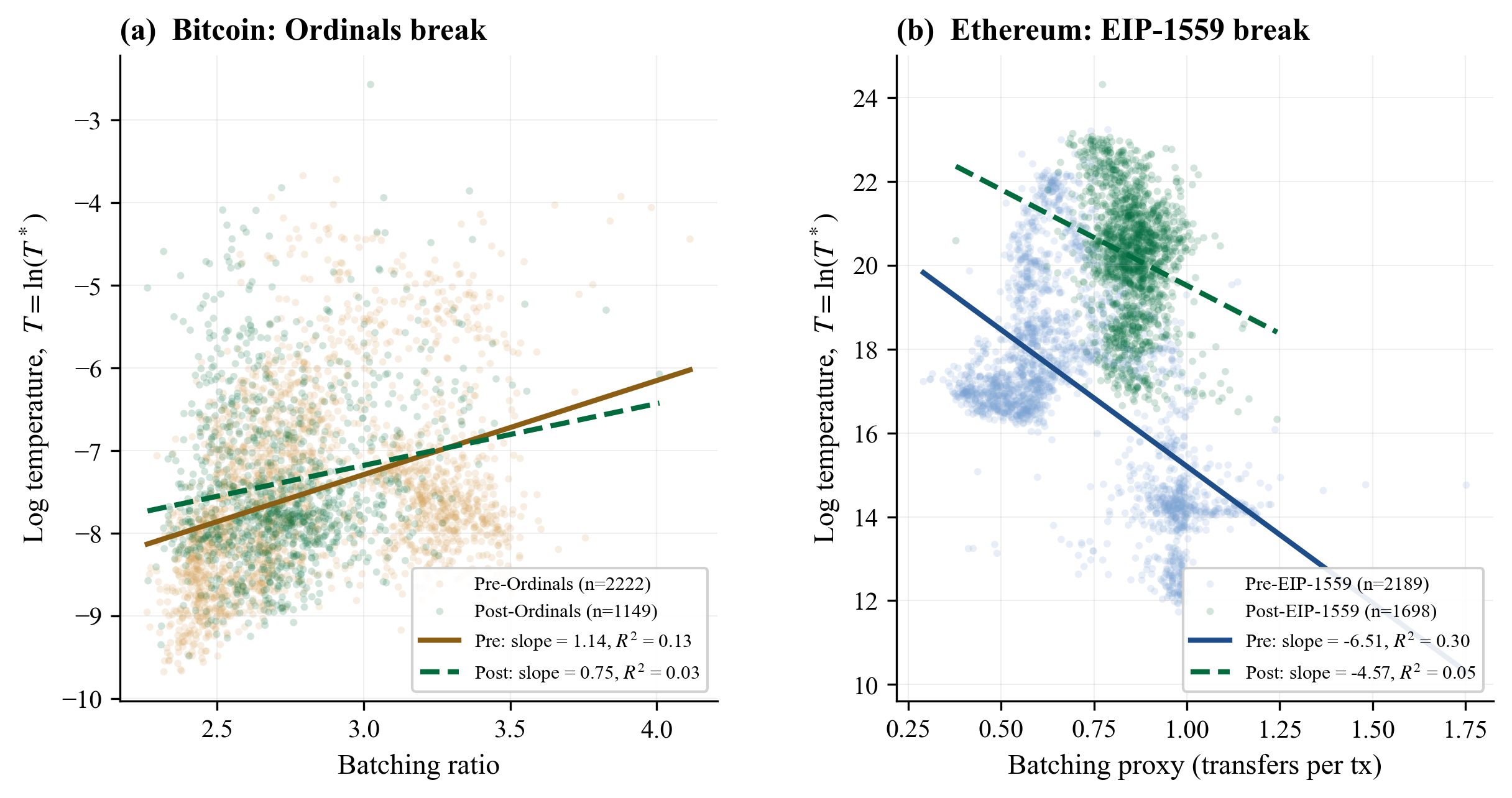

Supplement: Supplementary file 1 [file entropy-28-00508-s001.zip › entropy_submission_entropy_optimized/figures/fig5_structural_breaks.png]

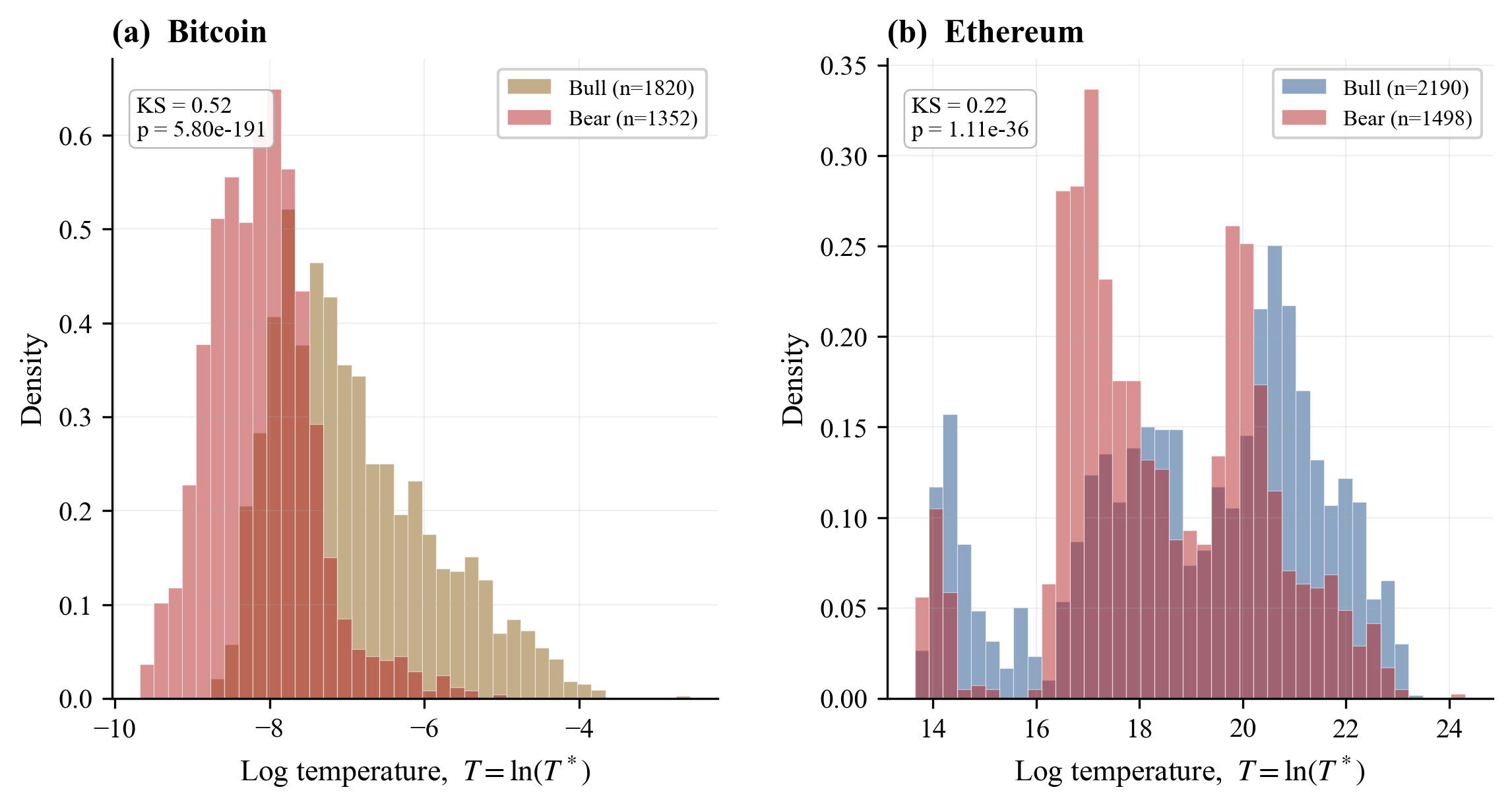

Supplement: Supplementary file 1 [file entropy-28-00508-s001.zip › entropy_submission_entropy_optimized/figures/figS1_bull_bear_distributions.png]
